# The mechanism of transactivation regulation due to polymorphic short tandem repeats (STRs) using IGF1 promoter as a model

**DOI:** 10.1038/srep38225

**Published:** 2016-12-02

**Authors:** Holly Y. Chen, Suk Ling Ma, Wei Huang, Lindan Ji, Vincent H. K. Leung, Honglin Jiang, Xiaoqiang Yao, Nelson L. S. Tang

**Affiliations:** 1 Department of Chemical Pathology, Faculty of Medicine, The Chinese University of Hong Kong, Shatin, Hong Kong SAR, China; 2 Department of Psychiatry, Faculty of Medicine, The Chinese University of Hong Kong, Shatin, Hong Kong SAR, China; 3 State Key Laboratory of Bioactive Substance and Function of Natural Medicines, Department of Pharmaceutics, Institute of Materia Medica, Chinese Academy of Medical Sciences and Peking Union Medical College, Beijing, China; 4 Department of Biochemistry and Molecular Biology, Zhejiang Provincial Key Laboratory of Pathophysiology, Ningbo University School of Medicine, Ningbo, China; 5 Department of Animal and Poultry Sciences, Virginia Polytechnic Institute and State University, Blacksburg, Virginia 24061, USA; 6 School of Biomedical Sciences, The Chinese University of Hong Kong, Hong Kong, China; 7 Laboratory of Genetics of Disease Susceptibility, Li Ka Shing Institute of Health Sciences, The Chinese University of Hong Kong, Shatin, Hong Kong SAR, China; 8 Functional Genomics and Biostatistical Computing laboratory, Shenzhen Research Institute, The Chinese University of Hong Kong, China; 9 KIZ/CUHK Joint Laboratory of Bioresources and Molecular Research in Common Diseases, Kunming, China

## Abstract

Functional short tandem repeats (STR) are polymorphic in the population, and the number of repeats regulates the expression of nearby genes (known as expression STR, eSTR). STR in IGF1 promoter has been extensively studied for its association with IGF1 concentration in blood and various clinical traits and represents an important eSTR. We previously used an *in-vitro* luciferase reporter model to examine the interaction between STRs and SNPs in IGF1 promoter. Here, we further explored the mechanism how the number of repeats of the STR regulates gene transcription. An inverse correlation between the number of repeats and the extent of transactivation was found in a haplotype consisting of three promoter SNPs (C-STR-T-T). We showed that these adjacent SNPs located outside the STR were required for the STR to function as eSTR. The C allele of rs35767 provides a binding site for CCAAT/enhancer-binding-protein δ (C/EBPD), which is essential for the gradational transactivation property of eSTR and FOXA3 may also be involved. Therefore, we propose a mechanism in which the gradational transactivation by the eSTR is caused by the interaction of one or more transcriptional complexes located outside the STR, rather than by direct binding to a repeat motif of the STR.

Genetic variations in gene promoters play key roles in the determination of gene expression and phenotypes, including disease predisposition. Single nucleotide polymorphisms (SNPs) are the most commonly studied genetic variations and have been considered as the primary functional element in phenotype determination. The alternate alleles of a SNP in a gene promoter may result in either the formation or abolition of a binding site for transcription factors (TFs) and are therefore believed to play piloting roles in the transactivation of gene expression and quantitative trait loci^1^,^2^,^3^,^4^. In contrast, another type of common genetic variation, short tandem repeats or microsatellites (STRs) has been considered functionally neutral, as it alters only the length of DNA segments via repeat sequences. A STR consists of repeating units of a motif ranging from 2 to 13 base pairs (bps)^5^,^6^. With the exception of disease causing massive expansion of trinucleotide repeats, little biological evidence has yet been found that STRs can regulate gene transactivation, and most data come from studies of primitive eukaryotes^7^,^8^,^9^,^10^. In human genetics, they are considered biologically inert genetic markers and have been used exclusively in forensic applications and linkage analysis^11^.

Recently, a renewed interest in the functional role of STRs has developed. First, STRs are abundantly located in promoters across eukaryotic organisms (ranging from yeast to rodents and humans)^12^,^13^,^14^ and variations in the length of a few STRs in model organisms have been associated with changes in phenotype or gene transactivation^14^,^15^,^16^. Second, an increasing body of evidence suggests that STRs play important roles in molecular evolution^17^. Sonay *et al*. studied STRs in both human and nonhuman great ape genomes and showed that STRs contribute significantly to the diversity and divergence of gene expression among species^13^,^18^,^19^,^20^. These findings indicate that STRs are essential elements in molecular evolution and underscore their functional potential. Third, a considerable number of associations have been found between variation in STRs and in human phenotypes^6^,^15^,^21^, and STR have been suggested to account for the “missing heritability” in genome-wide association studies.

The renewed interest in STRs, particularly their functional potential and role in human diseases, has led to the development of tools and catalogs of STRs. For example, Willems *et al*.^5^ used the 1000 Genomes sequence data to reveal the allelic spectrum of 700,000 STRs among 1000 individuals sampled worldwide. Dinucleotide repeats were the most abundant STR variation in the genome^5^. New tools have also been developed to give reliable call of STR alleles from high-throughput sequencing data^5^,^22^,^23^. As a consequence, more comprehensive catalogs of STRs in the human genome have become available^5^,^24^,^25^. Recent findings by Gymrek *et al*. confirmed the presence of more than 2000 functional STR loci that are correlated with gene expression, which are termed expression STRs (eSTRs)^26^,^27^.

Although evidence to support STRs as functional elements continues to accumulate, little information is known about how they operate. The only difference between any two STR alleles is the number of repeats; there is no change in the nucleotide base as in the case of SNPs, which could be responsible for alterations in the binding sites for nuclear proteins and transcriptional factors. The most abundant STRs are dinucleotide repeats, and the difference between alleles could be as little as lengthening (or shortening) by 2 bps. Furthermore, these changes in length (i.e. number of repeats) occur within a sequence of identical 2-bp repeating motifs; given such minute alterations, it is difficult to envision how eSTRs may regulate gene function.

We have studied the transactivation mechanism of STRs using IGF1 as a model for other dinucleotide repeats. Our early results were consistent with genetic epidemiological findings and supported a biological basis for the association between the blood concentration of IGF1 and the alleles of the STR in IGF1 promoter^28^,^29^. Near the transcription starting site, the promoter of IGF1 contains three tagging SNPs and one STR (a CA dinucleotide repeat) within a common haploblock^28^. We previously showed that the number of repeats of this STR is inversely correlated with reporter gene transactivation using an *in-vitro* luciferase model. Interestingly, this gradational transactivation property was only found in one of the two prevalent SNP haplotypes. In this study, we used an *in-vitro* reporter assay to study and localize the functional elements within this promoter and to investigate the mechanism of the gradational effect of microsatellite length on transactivation. We approached this question at two levels. First, at the genomic DNA level, we used serial deletion fragments of the promoter to localize functional units in the promoter. Second, we identified which transcriptional factors (TFs) were involved through a series of experiments by removing each TF from the expression plasmid mix. The results of this two-tiered experimental approach provided new insights into the gradational transactivation property of these microsatellites, which may represent a common mechanism of transactivation regulation shared with other eSTRs.

## Methods

### Allelic frequencies of different populations and population differentiation

Genotype data for these three SNPs and their neighboring SNPs were obtained from the HapMap database, and their fixation index (*F*_ST_) values were calculated according to the equation described by Weir and Cockerham^30^. The haplotype frequencies were based on HapMap data of Chinese, Japanese and Caucasian. The three SNPs that were further investigated by luciferase reporter experiments are shown in capital letter in the haplotypes.

### Construction of plasmids

**Figure 1:**
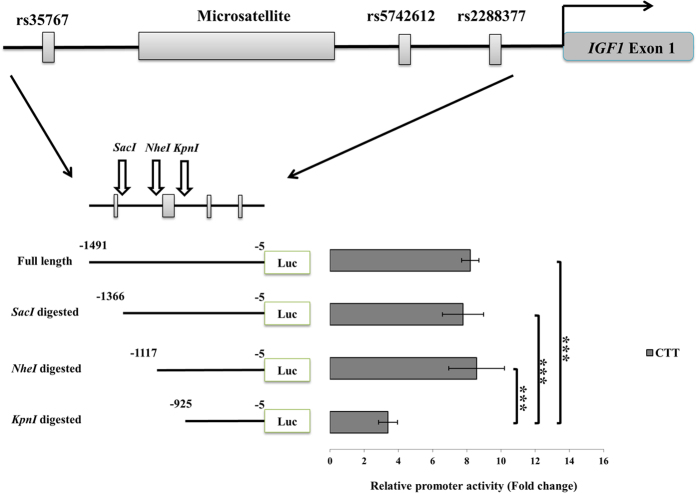
Effect of serial 5′ deletion of IGF1 promoter on transcriptional activity. We compared the luciferase activity of IGF1 promoter fragments with different lengths. A schematic figure of IGF1 promoter fragment is shown at the top. Relative positions of the tagging genetic variants and restriction sites are indicated by rectangles and arrows, respectively. The full length and digested fragments cloned into the 5′-end of luciferase gene of pGL4.10 vector are represented by lines below the schematic figure of IGF1 promoter fragment. The number on the left of the lines indicates the start site relative to the translation start site (TSS) of IGF1, while the number on the right indicates the end site relative to TSS. The relative promoter activity is shown as fold change compared to an empty pGL4.10 vector. Length of bar indicates the mean, and error bars indicate the standard deviation of four independent experiments, each of which consisted of four replicates. Data were analyzed by one-way ANOVA (three or more groups), followed by Tukey’s test as a post hoc test (***p < 0.005 by post hoc test).

A schematic view of the structure of IGF1 promoter is shown in Fig. 1. For reporter constructs of the common promoter haplotypes (Supplementary Table T1)^29^, 1.5-kb long fragments of the IGF1 promoter region, ranging from −1491 to −5 relative to the translation start site, were synthesized by PCR using genomic DNA from normal subjects. The fragment was amplified by a forward primer containing a *BglII* restriction site (underlined) (5′-AGCAGATCTGCCCCAGGATAACACAAAGA-3′) and a reverse primer containing a *HindIII* restriction site (underlined) (5′-AGCAAGCTTGCTTCTGAAGTACAAAGTCT-3′). The PCR products were purified using the Wizard SV *Gel* and PCR *Clean*-Up System (Promega, Madison, WI) according to the manufacturer’s instructions and digested by *BglII* (NEB, Ipswich, MA) and *HindIII* (NEB, Ipswich, MA). The gel-extracted products were purified as described above and cloned into the *BglII* and *HindIII* sites of a promoter-less *firefly* luciferase vector, pGL4.10 (Promega, Madison, WI). Reporter constructs with uncommon promoter haplotypes (Supplementary Table T1), were obtained by *in vitro* hybridization of constructs with common promoter haplotypes, as previously described^31^. For reporter constructs of serial deletion fragments, constructs with a full-length IGF1 promoter fragment were used as a template and sequentially digested by *SacI, NheI* and *KpnI* (NEB, Ipswich, MA) (Supplementary Fig. F1). Expression plasmids of key regulators in the GH-IGF1 axis, pcDNA3-hGHR and pcDNA3-hFOXA3, which constitutively express human GHR and human FOXA3, respectively were constructed as previously described^32^. Expression plasmid of pSX-hStat5B, which constitutively expresses human signal transducer and activator of transcription 5B (Stat5B), was a kind gift from Dr. W. J. Leonard (Laboratory of Molecular Immunology, National Institutes of Health). All constructs were verified by sequencing (BGI, China).

### Cell line and culture

The human lung epithelial cell line Beas-2B was obtained from American Type Culture Collection (ATCC, Manassas, VA). The cell line was maintained in Dulbecco’s Modified Eagle Medium (DMEM)/F12 (Life Technologies, Grand Island, NY), supplemented with 10% heat-inactivated fetal bovine serum (Life Technologies, Grand Island, NY). Cells were incubated at 37 °C with 5% CO_2_. This cell line was selected due to the previous finding that it was a suitable cell model for manipulation of TFs related to IGF1 promoter^29^.

### Transient transfection and dual-luciferase assay

The cells were seeded in 24-well plates at a density of 5 × 10^4^ 24 hours before transfection. Cell transfection was performed using X-tremeGene HP (Roche, Indianapolis, IN), according to the manufacturer’s protocol. For each well, 0.5 μg of the reporter construct was transfected along with 1ng of cytomegalovirus (CMV)-controlled *Renilla* luciferase vector pGL4.75 (Promega, Madison, WI), which was used to adjust for transfection efficiency, and 0.5 μg of pcDNA3-hGHR, 0.25 μg of pcDNA3-hFOXA3 and 0.25 μg of pSX-hStat5B, which were used to activate the promoter fragments. The transfected cells were incubated for 48 hours before they were harvested for luciferase assay. The activity of *firefly* and *Renilla* luciferase was measured by a Dual Luciferase Reporter Assay System (Promega, Madison, WI). For each reaction, 50 μl of LARII and 50 μl of Stop & Glo Reagent were added to 10 μl of cell lysate. The output signal was detected with a Victor *X*3 Multilabel Plate Counter (PerkinElmer, Turku, Finland). The *firefly* luciferase activity encoded by the promoter-reporter construct was normalized to the *Renilla* luciferase activity encoded by CMV promoter to control for variation in transfection efficiency. All assays were performed in four independent experiments, each of which consisted of four replicates for each haplotype (n = 16). Coefficient of variation (CV) was less than 25% for each group.

### Statistical analysis

Population differentiation was determined by classic *F*_ST_ analysis^30^. The data from the luciferase assays were analyzed with SPSS 16.0.2 (SPSS Inc., Armonk, NY). Student’s t test was used to compare the means between two groups. For multiple group comparison, one-way ANOVA was used to compare the means among groups and Tukey’s test was used as a post-hoc test. All data were expressed as the means ± SD. Differences for which the p-value was less than were considered to indicate statistical significance.

## Results

### Molecular evolution analysis of IGF1 promoter

The allelic distribution of these three studied SNPs (rs35767:T > C, rs5742612:T > C and rs2288377:T > A) differed significantly between Caucasians and Asians (Table 1). The genotypes of reported IGF1 SNPs were obtained from the HapMap database to construct the phased haplotypes for Asians (Chinese and Japanese) and Caucasians (Table 2). The most common haplotype found in both populations were gggCTTac, for which the frequencies for Asians and Caucasians were 66% and 88%, respectively. However, the second-most common haplotype found in Asians, atcTCAga, was only found in low percentage of Caucasians (2%). For the haplotype of the three SNPs studied here, there was a large difference between Asians and Caucasians. The haplotype CTT was found in 90% of Caucasians but in only 69% of Asians. In contrast, the haplotype TCA appeared to be specific to Asians (27%) and was rare in Caucasians (2%).

This analysis suggested that significant differences in haplotype structure were present among the different populations. Fixation index (*F*_ST_), measures the differentiation of a subpopulation relative to the total population and is directly related to the variance in allele frequency among subpopulations. A high *F*_ST_ implies a high level of differentiation. Genotype data for these three SNPs and their neighboring SNPs were obtained from HapMap database and *F*_ST_ was calculated according to equation described by Weir and Cockerham^30^. The analysis showed that rs35767 has the lowest *F*_ST_ of among the three SNPs, with *F*_ST_ = 0.025. For rs5742612 and rs2288377, the *F*_ST_ was 0.346 and 0.387, respectively. The low *F*_ST_ of rs35767 suggests that this is a universal allele, distributed across the world’s population, and may have an important biological function.

### What elements are necessary for the gradational transactivation found among 17, 18, 19, 21 (CA) repeats on the C-T-T haplotype background?

Our previous study showed that various STRs with lengths 17, 18, and 19 repeats on the background of the common haplotype C-T-T (i.e. C17TT, C18TT and C19TT) had significantly different transcription activity^29^. To localize the promoter segments responsible for this gradational transcriptional activity of the haplotype C-T-T, we performed a serial 5′-end deletion assay on the full-length promoter of the C-21-T-T haplotype, which was prepared by *in-vitro* mutagenesis^29^.

Serial deletion mutants were prepared from −1491 to −925 on the construct with haplotype C-T-T (Fig. 1). The luciferase activity of the full-length promoter was up to 8-fold higher than that of the empty vector, and the promoter activity remained unaffected by the deletions up to a fragment including −1117 bp (a 374-bp deletion). Further removal of a 192-bp segment covering the site of the microsatellite, which was from −1117 bp to −925 bp, significantly reduced the promoter activity to only 3 times that of the empty vector (a 62.5% decrease). This result suggested that the microsatellite might play a role in regulating of the promoter activity.

### The 125-bp (−1491 to −1366) segment is required for the gradational transactivation among various microsatellite repeats

In our previous study, we identified a gradational effect of the microsatellite in the haplotype C-T-T, in which promoters with a longer microsatellite had a lower transcriptional activity^29^. In the 125-bp long interval of the promoter region from −1491 to −1366, a location immediately upstream of the microsatellite, there was only one common genetic variation, which was a SNP, rs35767. To investigate whether the 125-bp segment (containing rs35767) is crucial for the gradational effect of the microsatellite, we removed the segment by *SacI* digestion and compared the transcriptional activity of the full-length and digested (without the 125 bp) promoter fragments.

**Figure 2:**
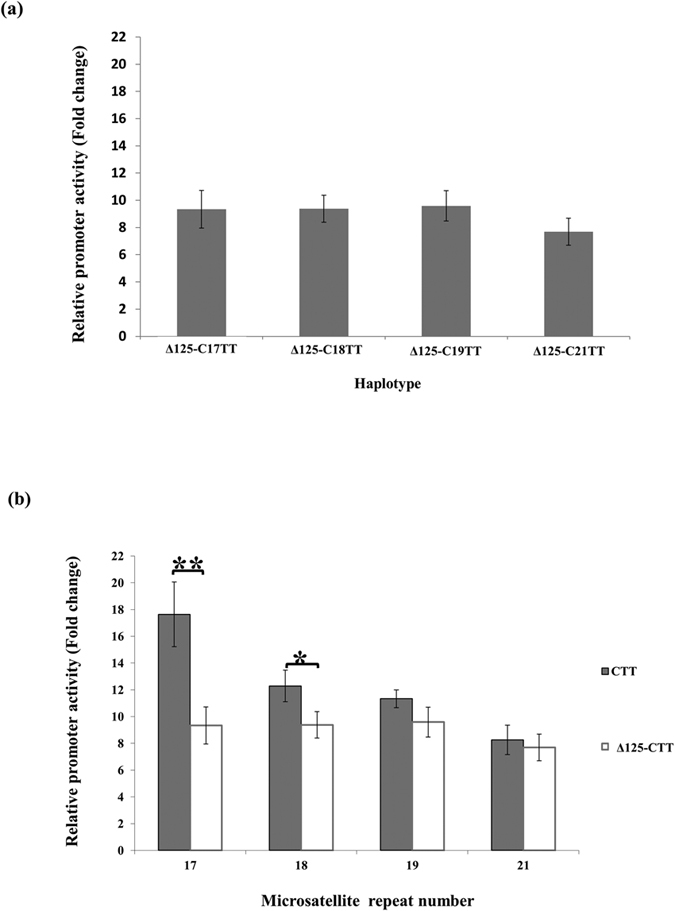
Effect of the 125-bp (−1491 to −1366) segment on IGF1 promoter activity. **(a)** After removal of the 125-bp segment located upstream of the STR, there was no significant difference among promoter fragments with different microsatellite repeat numbers. **(b)** When compared to full-length promoter fragments^29^, removal of the fragment resulted in significant decrease of promoter activity in plasmids carrying low microsatellite repeat numbers (17 or 18 repeats). Relative luciferase activity is shown as mean ± SD. One-way ANOVA was used for group comparison (in **a**) and student’s t test was used for comparison of same STR repeat of two haplotypes (in **b**). Each assay was repeated four times in each of the four independent experiments (n = 16) (*p < 0.05; **p < 0.01 by student’s t test).

As shown in Fig. 2a, after deletion of the 125-bp segment, there was no difference in transcriptional activity among promoters of different microsatellite lengths. These results indicated that this 125-bp upstream segment (with the C allele of rs35767) was necessary for the gradational promoter transcriptional activity among different microsatellite lengths. This conclusion was in line with the findings of our previous study, when comparing the digested fragments to full-length promoter fragment (Fig. 2b)^29^, and removal of the 125-bp segment led to a decrease of promoter activity with short STRs, i.e. 17 or 18 repeats. Specifically, deletion of the 125-bp segment resulted in a 44% decrease of promoter activity when the microsatellite had 17 repeats, but only a 25% decrease when the microsatellite had 18 repeats.

### Gradational transcriptional activation ability of the 125-bp (−1491 to −1366) segment is specific to the C allele of rs35767, a putative binding site for C/EBPD

It has been reported that a C/EBPD transcription activation complex binds exclusively to the C allele of rs35767 to activate IGF1 promoter activity^33^. To test whether the C allele is responsible for the promoter activation ability of the 125-bp segment and the length effect of the microsatellite, we mutated haplotype C-T-T to haplotype T-T-T in all of the promoter fragments and examined for their gradational transactivation properties as a function of microsatellite repeat length.

**Figure 3:**
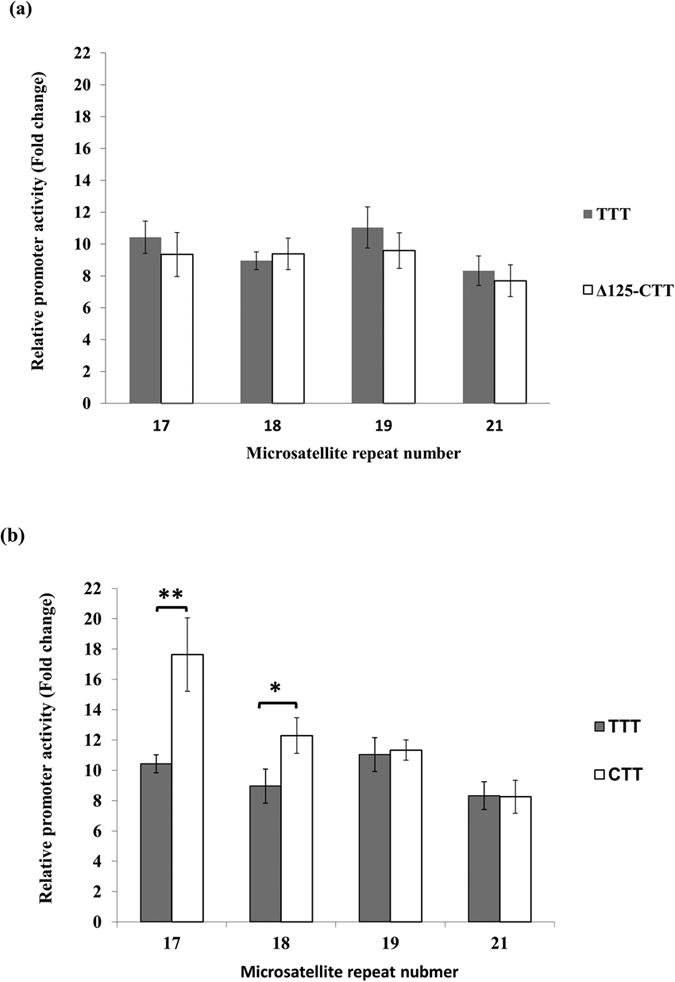
Effect of the C allele of rs35767 on IGF1 promoter activity. **(a)** There was no significant difference in promoter activity between haplotype T-T-T and *SacI* digested haplotype C-T-T. **(b)** Compared to the promoter activity of haplotype C-T-T, which has been described previously^29^, a significant decrease in promoter activity was observed in plasmids with low microsatellite repeat numbers, 17 or 18 repeats. Relative luciferase activity is shown as mean ± SD. One-way ANOVA was used for group comparison among four STRs (in **a**) and student’s t test was used for comparison of two haplotypes (in **b**). Each assay was repeated for four times in each of the four independent experiments (n = 16) (*p < 0.05; **p < 0.01 by student’s t test).

On the background of haplotype T-T-T, the gradational transactivation effect of different microsatellite lengths was abolished (Fig. 3a), indicating that the microsatellite length no longer had an effect on the promoter activity. To determine whether rs35767 C/T is primarily responsible for the necessary role of the 125-bp segment, we compared the promoter activity between haplotype T-T-T (the full-length) and haplotype C-T-T with 125 bp segment deleted (Δ-125 CTT). For all microsatellite lengths, there was no difference between the full-length T-T-T and the deletion fragments, indicating that the C allele is responsible for the transcriptional property of the fragment (Fig. 3a). Compared with the full-length promoter fragments in haplotype C-T-T^29^, a level-off effect of transactivation was observed particularly among short microsatellite (17 or 18 repeats) (Fig. 3b).

### Gradational transactivation is dependent on the expression of another transcriptional factor FOXA3

Because rs35767 is located more than 1000 bp upstream of IGF1, it may interact with another TF close to the transcription start site of IGF1 to recruit transcription machinery and activate gene expression^34^. In the liver, where circulating IGF1 is produced, one common interaction partner of C/EBPD is FOXA3^35^, whose putative binding site is predicted to be downstream of the STR (using Promo software^36^). To investigate the effect of FOXA3 on a promoter with an intact C/EBPD binding site (the C allele of rs35767), we replaced the expression vector of FOXA3 with an equal amount of empty control vector in the luciferase system to compare the promoter activity of the full-length haplotypes (C-T-T).

**Figure 4:**
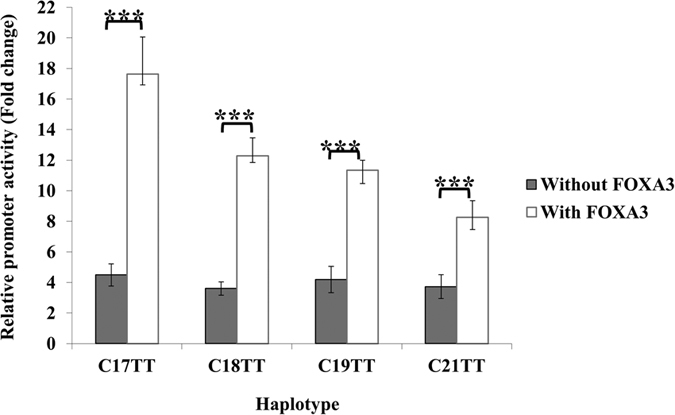
Effect of FOXA3 on IGF1 promoter activity. In the absence of FOXA3, there was no gradational transactivation among promoter fragments with different microsatellite lengths. In the presence of FOXA3, the length of microsatellite repeats was significantly associated with the transcriptional activity, the lower the repeat numbers, the higher the transcriptional activity. Relative luciferase activity is shown as mean ± SD. Student’s t test was used for comparison of two haplotypes. Each assay was repeated four times in each of the four independent experiments (n = 16) (***p < 0.001 by student’s t test).

In the absence of FOXA3, gradational transactivation as a function of microsatellite with different lengths in haplotype C-T-T was abolished, and thus there was no relationship between microsatellite length and promoter activity (Fig. 4). Promoters of different microsatellite lengths showed a roughly uniform transactivation capacity, they had a value ~4 times that of the empty vector, with no significant difference among them. Although the transcriptional activity was significantly lower in the absence of FOXA3, the levels were high enough to discern any gradational transactivation among STR alleles if it was present.

## Discussion

The association between genetic variations in IGF1 promoter and inter-individual variation in circulating IGF1 levels and disease susceptibility has long been identified in epidemiology studies, but the underlying mechanism has not been elucidated^37^,^38^,^39^,^40^. We previously showed that the major regulatory unit in IGF1 promoter is the haplotype, in which tagging SNPs interact with the microsatellite in the regulation of IGF1 expression^28^,^29^. In the conventional understanding of human genetics, STRs are recognized as the sole neutral genetic markers. This view is widely accepted given that variation in the repeat length is apparently trivial addition of 2 bp motifs. Although functional STRs were previously reported only in primitive organisms and lower eukaryotes^7^,^10^, recent reports of eSTR in humans shed light on the importance of this long-neglected genetic variation. The unusual linkage of SNP and STR in IGF1 promoter and the recent interest in eSTR prompted us to examine the function of this STR.

The *in-vitro* luciferase reporter model used here had been developed earlier, and the optimization procedure was described in more detail in our previous publication (Chen, *et al*.^29^). In brief, we selected a cell line with minimal endogenous IGF1 expression to avoid interference from endogenous transcription regulation mechanisms. Furthermore, the absent or low expression of related TFs in the cell model provides the opportunity for manipulation using expression plasmids. The amount of expression plasmids used had been optimized to reach a plateau response. However, as the experiment was performed *in-vitro*, we cannot be certain whether the findings also hold under physiological (*in-vivo*) conditions, which is the essential limitation of all *in-vitro* studies.

To investigate the underlying mechanism of the differential transcriptional activities between haplotypes C-T-T and T-C-A, a 5′-serial deletion experiment was performed to analyze the effect of various segments of IGF1 promoter on transcriptional activity. We found that a 125-bp segment, in which rs35767 was located, and a 925-bp segment, in which rs5742612 and rs2288366 were located, contributed to the differential transcriptional activities between the two haplotypes. This indicates that functional elements are present inside these two promoter segments which enable the eSTR property.

SNP rs35767 located in the 125-bp segment, is a recognized functional SNP^33^. A transcriptional activator C/EBPD complex binds exclusively to the C allele of this SNP and activates promoter transcriptional activity^33^. Moreover, genome-wide association studies and epidemiologic studies have consistently demonstrated a significant association between this SNP and circulating IGF1 levels or IGF1-related phenotypes^41^,^42^,^43^,^44^,^45^. Therefore, there is strong evidence for the role of rs35767 in the regulation of IGF1 promoter activity. Here we investigated the role of this SNP in the eSTR property of this promoter. We previously identified a length effect of the IGF1 microsatellite (i.e. eSTR) exclusively in haplotype C-T-T, in which longer microsatellites had lower transcriptional activity^29^. Here, we showed that this gradational effect of the eSTR depended on the C allele of rs35767.

Furthermore, our results suggested that the importance of FOXA3 for gradational transactivation. FOXA3 is a liver-enriched TF and a known interaction partner of C/EBPD complex in the liver^33^,^35^. It is also crucial for the expression of IGF1 in other mammals^32^. Replacement of the FOXA3 expression vector by an empty vector in the system significantly decreased the promoter transcriptional activity and nullified the gradational effect of the microsatellite length.

We confirmed the population differentiation in variations of IGF1 promoter, e.g. rs35767, using population genetics and molecular evolution analysis. Our results suggested that rs35767 had the lowest *F*_ST_ among the neighboring SNPs. The mean genome-wide value of *F*st across the population for all 2.8 million Phase II HapMap SNPs is 0.11. Assuming neutrality, all variants were similarly affected by only human demographic history. However, the significantly low population differentiation indicated by its *F*st suggests a potentially strong natural selection pressure upon this IGF1 promoter SNP. On the basis of population genetic studies, it has been suggested that value of *F*_ST_ < 0.05 can be regarded as low, and some examples of SNPs with low *F*_ST_ are associated with disease susceptibility^46^. This observation further supports the importance of rs35767 in modulating the transactivating ability of IGF1 promoter.

**Figure 5:**
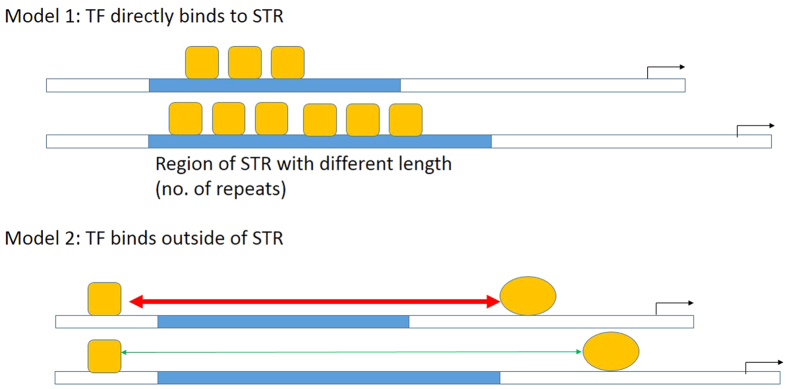
Schematic diagrams of two alternate hypothetical models for molecular action of STR of different length. In Model 1, transcriptional factor (TF) binds directly to the repeat motifs of STR. Therefore, the longer allele of the STR allows the binding of more TF and thus results in higher (or lower if that TF is inhibitory) transactivation activity. In Model 2, one or more TF(s) bind outside the STR. The steric interaction between TFs bind to both ends of the repeat motif provide the basis of differential transactivation activity between different STR alleles.

**Figure 6:**
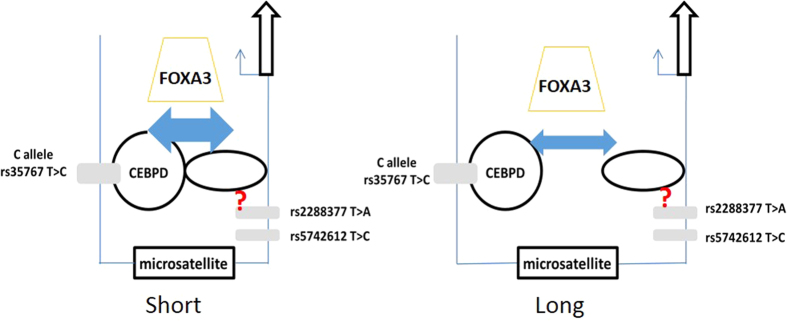
Putative model of the regulation of IGF1 promoter activity by the microsatellite and SNPs. The arrow indicates the transcription start site of IGF1. Genetic variants in this study are depicted by rectangles. Transcription factor C/EBPD complex is indicated by circles. When the microsatellite length is short, the interaction between transcriptional complexes across the STR may be stronger than the case when microsatellite length is long. C/EBPD complex and FOXA3 may be involved in this interaction. The SNP rs35767 accounts for most of the transactivation property in the segment upstream of the microsatellite, while the exact functional location downstream of STR is not certain^29^,^33^.

Despite the recent genomic scale discovery of more than 2000 eSTRs, little is known about the mechanism on how the length of the microsatellite affects gene expression. There are two potential models for the operation of eSTR (see Fig. 5). Model 1: Some TF binds directly to the microsatellite and the length of the microsatellite determines the extent of this binding and thus the transactivation capacity. Model 2: (as an alternative to model 1) TF do not bind directly to the microsatellite. Instead of direct binding between TFs and the STR, one or more TFs bind outside the STR. The length of the STR determines the intensity of interaction between the TF complexes and thus results in a gradational transactivation. As the C/EBPD complex is located upstream of the microsatellite, our data provide strong support for Model 2 (Fig. 6). FOXA3 may be involved, and a putative binding site was predicted downstream of the STR by the bioinformatics program. However, we have not confirmed by experiment whether this is a functional binding site.

In conclusion, this study provides support for a model of the mechanism of eSTR based on cooperation between the STR and adjacent TF binding complexes. Our data, together with the results of our previous epidemiological and functional studies, demonstrate that the eSTR effect relies on both the microsatellite and the SNPs. In addition, our results suggest a novel regulatory mechanism for microsatellites in humans.

## Conclusions

To investigate the regulatory mechanism of eSTR, we performed *in vitro* reporter assays to compare the transcriptional activity of IGF1 promoter fragments with various STR lengths and allelic compositions. We identified two regions outside the STR that contribute to the distinct regulatory mechanisms of haplotype C-T-T and T-C-A: a 125-bp segment with a functional SNP, rs35767, and a 925-bp segment with two common SNPs, rs5742612 and rs2288377. In the haplotype C-T-T, an eSTR effect was found in which higher transactivation was correlated with shorter STRs. The eSTR property is dependent on the C/EBPD complex, which binds upstream to the microsatellite. This led us to suggest a model for eSTR function involving the binding of one or more TF in the vicinity of the microsatellite but not directly onto the repeat motifs.

## Additional Information

**How to cite this article**: Chen, H. Y. *et al*. The mechanism of transactivation regulation due to polymorphic short tandem repeats (STRs) using IGF1 promoter as a model. *Sci. Rep.* **6**, 38225; doi: 10.1038/srep38225 (2016).

**Publisher's note:** Springer Nature remains neutral with regard to jurisdictional claims in published maps and institutional affiliations.

## Supplementary Material

Supplementary Information

## Figures and Tables

**Table 1:** Allelic frequencies and *F*_ST_ in the worldwide population for SNPs that are important in modulating the transcriptional activity of IGF1*.

	Frequencies of reference allele		*F*_ST_
	CEU	CHB	
rs35767	0.115	0.354	0.025
rs5742612	0.978	0.704	0.346
rs2288377	0.977	0.739	0.387

^*^CEU represents HapMap Caucasians, Asian include both CHB represents HapMap Chinese and Japanese. *F*_ST_ values was calculated for 4 populations in HapMap.

**Table 2:** Table showing reported IGF1 promoter haplotype frequencies in Asians (Chinese and Japanese) and Caucasians*.

*IGF1* haplotype#	Haplotype Frequencies‡	
	CHB + JAP	CEU
CTT		
gggCTTac	66%	88%
gggCTTaa	1%	0%
gggCTTgc	1%	1%
gggCTTga	1%	0%
aggCTTac	0%	1%
subtotal	69%	90%
TCA		
atcTCAga	22%	2%
atcTCAac	3%	0%
atcTCAgc	2%	0%
subtotal	27%	2%

^*^Individual haplotypes were obtained from the HapMap database.

^#^Haplotypes were composed of the following SNPs of IGF1 in listed order: rs17032648, rs12579108, rs12579077, **rs35767**, **rs5742612**, **rs2288377**, rs2162679, rs5742615 (SNPs in bold were investigated in this study).

^‡^CHB represents HapMap Chinese population, JAP represents HapMap Japanese population, and CEU represents HapMap Caucasians.
