# Supplementary material for: The mechanism of transactivation regulation due to polymorphic short tandem repeats (STRs) using IGF1 promoter as a model
Source: Sci Rep. 2016 Dec 2;6:38225. doi: 10.1038/srep38225 (PMC5133613; doi:10.1038/srep38225)
Supplement: Supplementary Information [file srep38225-s1.pdf]

The mechanism of transactivation regulation due to polymorphic short tandem repeats (STRs) using *IGF1* promoter as a model

Holly Y Chen[1,ǂ,#], Suk Ling Ma[2,ǂ], Wei Huang[3], Lindan Ji[4], Vincent HK Leung[1], Honglin Jiang[5], Xiaoqiang Yao[6], Nelson LS Tang[1,6,7,8,9*]

[1]Department of Chemical Pathology, Faculty of Medicine, The Chinese University of Hong Kong, Shatin, Hong Kong SAR, China.
[2]Department of Psychiatry, Faculty of Medicine, The Chinese University of Hong Kong, Shatin, Hong Kong SAR, China.
[3]State Key Laboratory of Bioactive Substance and Function of Natural Medicines, Department of Pharmaceutics, Institute of Materia Medica, Chinese Academy of Medical Sciences and Peking Union Medical College.
[4]Department of Biochemistry and Molecular Biology, Zhejiang Provincial Key Laboratory of Pathophysiology, Ningbo University School of Medicine, Ningbo, China.
[5]Department of Animal and Poultry Sciences, Virginia Polytechnic Institute and State University, Blacksburg, Virginia 24061, USA.
[6]School of Biomedical Sciences, The Chinese University of Hong Kong, Hong Kong, China.
[7]Laboratory of Genetics of Disease Susceptibility, Li Ka Shing Institute of Health Sciences, The Chinese University of Hong Kong, Shatin, Hong Kong SAR, China.
[8]Functional Genomics and Biostatistical Computing laboratory, Shenzhen Research Institute, The Chinese University of Hong Kong, China.
[9]KIZ/CUHK Joint Laboratory of Bioresources and Molecular Research in Common Diseases, Kunming, China.
[ǂ]These authors contributed equally to this work.
[#]Present address: Neurobiology-Neurodegeneration and Repair Laboratory, National Eye Institute, NIH, Bethesda, Maryland 20892, USA.

***To whom correspondence should be sent at the present address:**
Dr. Nelson Leung Sang TANG, Department of Chemical Pathology, Faculty of Medicine, The Chinese University of Hong Kong, Shatin, Hong Kong SAR, China
Phone: +852 26322320; Fax: +852 26365090; E-mail: nelsontang@cuhk.edu.hk.

Supplementary Information

Supplementary Table 1. Haplotypes of IGF1[a] promoter in the Chinese population of 169 normal subjects[b]

(A) Common haplotypes [c]

Haplotypes	Polymorphisms in the haplotypes			
	rs35767	Microsatellite	rs5742612	rs2288377
C17TT	C	17	T	T
C18TT	C	18	T	T
C19TT	C	19	T	T
T21CA	T	21	C	A

(B) Uncommon haplotypes[d] (most of them were constructed by in-vitro mutagenesis)

Haplotypes	Polymorphisms in the haplotypes			
	rs35767	Microsatellite	rs5742612	rs2288377
T17CA	T	17	C	A
T18CA	T	18	C	A
T19CA	T	19	C	A
C21TT	C	21	T	T
T17TT	T	17	T	T
T18TT	T	18	T	T
T19TT	T	19	T	T
T21TT	T	21	T	T

[a]IGF1 is short for insulin-like growth factor 1.

[b]The study subjects were described previously [23].

[c]Common haplotype is defined as a haplotype with frequency greater than 5%.

[d]Uncommon haplotype is defined as a haplotype with frequency less than 5%.

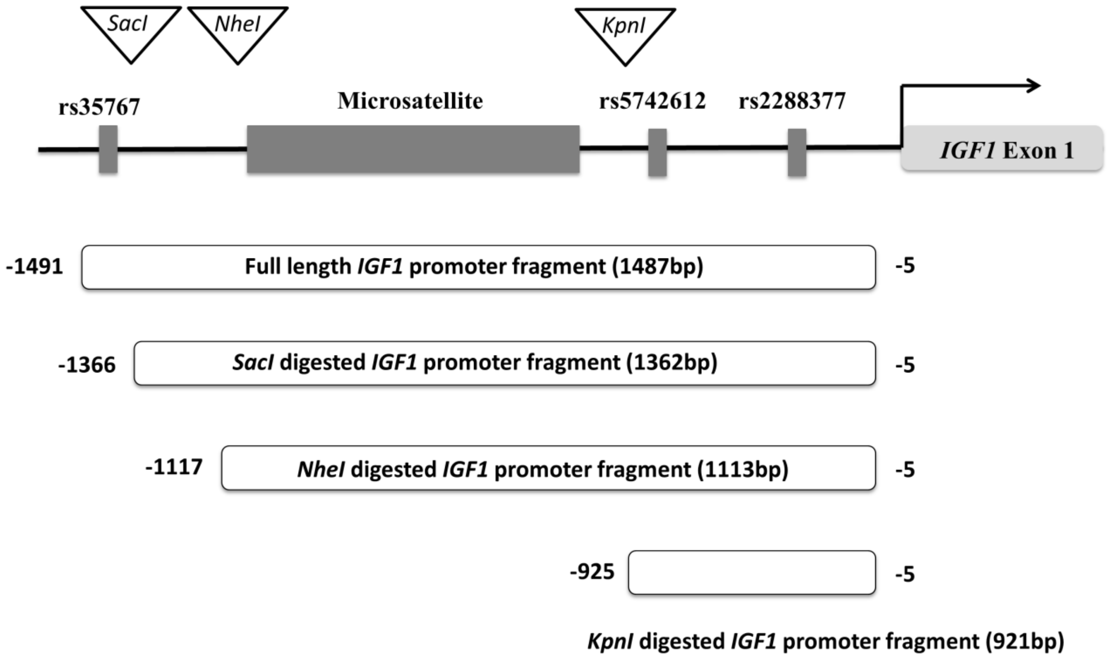

Supplementary Fig. 1 Serial 5' deletion of IGF1 promoter fragment. The horizontal line at the top of the figure depicts the IGF1 promoter region, and the arrow indicates the translation start site (TSS) of IGF1. The tagging genetic variants in this study are highlighted by rectangles, according to their relative positions in the promoter region. The triangles indicate the relative position of the restriction enzymes, *SacI*, *NheI* and *KpnI*. Bars at the lower part of the figure depict full length and digested promoter fragments. The number on the left of bars indicates the position of 5' end of fragments relative to TSS of IGF1, while the number on the right indicates the position of 3' end of fragments relative to TSS of IGF1.
